# Nixing the nightshades: Traditional knowledge of intoxicating members of the Solanaceae among hallucinogenic plant and mushroom users in Slovenia

**DOI:** 10.1371/journal.pone.0247688

**Published:** 2021-02-22

**Authors:** Karsten Fatur, Samo Kreft

**Affiliations:** Univerza v Ljubljani, Fakulteta za farmacijo, Ljubljana, Slovenia; University of Hyderabad, INDIA

## Abstract

Anticholinergic plants of the family Solanaceae have a long history of use as medicines, poisons, and recreational drugs. Though they were the intoxicating substances of choice throughout Europe for centuries, their use for these purposes has declined with the globalisation of other recreational drugs. The present study sought to examine the level of knowledge surrounding these plants among individuals who had used other hallucinogenic plants or mushrooms in Slovenia. Participants were questioned in regards to the anticholinergic Solanaceae that are known to grow wild in Slovenia: *Atropa belladonna* L., *Datura stramonium* L., *Hyoscyamus niger* L., and *Scopolia carniolica* L. As expected, only a small number of individuals had any substantial knowledge of these plants, and fewer still had used them; some were even unfamiliar with any of these plants. Knowledge of toxicity generally arose from family members, while books and the internet played prominent roles in regards to use knowledge. Knowledge of the plants was vastly varied, with many individuals confusing the plants for others, especially other members of the Solanaceae. Ultimately, a small group of individuals had the largest body of knowledge of these plants, though this was linked with university studies rather than traditional uses. Knowledge of the intoxicating Solanaceae has been largely lost in Slovenia among users of other botanical hallucinogens, likely due to the various dangers their use poses and the undesirable effects they often cause.

## Introduction

Perhaps best known for its range of useful crop plants such as tomatoes, potatoes, eggplants, and bell peppers, the Solanaceae is a widespread and popular botanical family. In addition to their uses for food, however, this family also contains plants that are rich in alkaloids that make them incredibly deadly. Among these plants, a small sub-group are known to be not only toxic, but also hallucinogenic.

These plants, sometimes called nightshades or hexing herbs, have been used in Europe for as long as history has been recorded, finding use as recreational substances [1–5], poisons and medicines [6–9], for hunting [10], in magic [11, 12], and in warfare [13, 14]; these have been extensively reviewed elsewhere [15]. These various uses are all linked to their alkaloid profiles, as these plants contain the anticholinergic tropane alkaloids hyoscyamine and scopolamine, which function as muscarine receptor antagonists; these receptors are found throughout the body’s smooth muscles, exocrine glands, and the nervous system [16]. As such, these compounds have a range of effects in the human body such as affecting heart rate and body secretions, as well as inducing a potentially dangerous state of delirium often accompanied by strong, realistic hallucinations [16, 17].

These cognitive effects allowed these plants to become important recreational substances throughout Europe, thus being employed to induce intentional altered states of consciousness for amusement; indeed, there are known to be surprisingly few native hallucinogenic plants in Europe, so though these plants are potentially deadly, they were widely employed [3, 18–21]. More specifically than just being hallucinogens, these plants belong to a sub-category known as deliriogens, known for their often dark and confusing mental effects [22, 23]. These effects are often undesirable and dangerous, and thus the various traditions of use for these plants are likely ceasing, with the associated knowledge of the plants disappearing as well. Indeed, previous research among Polish medical students showed a surprisingly low ability to recognise these plants [24].

That being said, in the modern day, a seemingly endless body of knowledge is available to anyone with the resources to connect to the internet. As access to this "novel global knowledge" has increased, local traditional knowledge seems to have come to play less of a role for many. The boundary between these two forms of knowledge, however, is perhaps not so clear. Though some would argue that ethnobotanical studies should seek to ascertain whether knowledge is traditional or newly gained through modern media, others would argue that the very notion of "traditional" knowledge as many of us hold it is fundamentally flawed in its manner of trying to pin the ever-changing face of knowledge systems down to a single static moment in time [25, 26]. Not only does each generation shape the world in which they live, but also the ways of knowing that they use. Globalisation and the vast availability of information in the modern era are themselves a sign of the times, and thus a part of the ever-evolving traditions of the present generations. As if this were not difficult enough to mentally sort, there are also issues as to what knowledge comes to be encoded in our cultures and become "traditional" while other types are neglected and relegated to oblivion [26]. The hybridisation of "traditional" knowledge systems with modern information has also played a role in certain ethnobotanical studies, further muddying the waters [27].

However, we can appreciate that the knowledge of today will be viewed as the tradition of tomorrow. Whether handed down by a shaman over generations or through a university professor or the internet, the knowledge that a group holds about certain plants speaks volume as to their importance and role within the culture. As such, even "new" knowledge is of importance within an ethnobotanical (and indeed more widely within an anthropological) framework.

As the plants presently discussed function as hallucinogens, it is also worth noting their use in ritual. Though they are known to have been used recreationally throughout Europe for centuries, very little knowledge about their use in ceremonial manners has been passed down, and thus their modern uses among pagan groups have required creative elements of reconstruction and novel innovation [28]. Indeed, a great deal of modern use for these plants can be traced to internet learning associated with individuals in various pagan communities [29]. Previous research on the ritual use of plants among modern pagan groups in Slovenia showed a high degree of knowledge being passed down between family members ("traditional" acquisition of knowledge), while hallucinogens used were often learned of from the internet and books ("non-traditional" acquisition of knowledge) [30]. As these "non-traditional" sources of knowledge come to be of greater importance, this must too be reflected in academic discourse and research.

The following article provides a glimpse into the world of varying and hybridised sources of knowledge through the aforementioned anticholinergic plants of the Solanaceae. Within Slovenia, these are *Atropa belladonna*, *Datura stramonium*, *Hyoscyamus niger*, and *Scopolia carniolica*. As new recreational drugs become available throughout Europe, it seems likely that the "traditional" knowledge of these plants and their uses are being forgotten, and in some cases, replaced. The present study sought to examine the level of and methods of inheriting knowledge that remains for these plants among individuals in the country who have previously used hallucinogenic plants and fungi.

## Methods

### Participant outreach

Participants who had previously used hallucinogenic plants and mushrooms were recruited through a combination of advertisements on social media and posters located throughout the capital city of Slovenia as well as through word of mouth. The interviews for the current study were connected with those for another project involving a general survey of the use of hallucinogenic plants and mushrooms in Slovenia, and as such, so too were the recruitment materials [31]. Posters and social media posts sought individuals with experience using hallucinogenic plants; anticholinergic Solanaceae plants were then brought up as a second stage of the interviews.

### Consent

In order to ensure informed consent, participants were told of the purpose of the study and how results would be shared, as well as that they would be able to retract their answers prior to publication should they wish it. Participants were informed that their names would be kept anonymous, and those being interviewed in person were asked if they were comfortable being audio recorded during the process. For those wishing to fill out electronic questionnaires, these were sent by email along with instructions. These questionnaires also served as the basis of the structured interviews that were given to the participants. Those to be interviewed in person gave consent verbally while those filling out the questionnaires did so implicitly in accordance with the submitting of the information.

Our institution does not have an ethical review board for ethnographic research, as it is not required. All research was, however, carried out in accordance with the principles set forth in the Declaration of Helsinki and to the highest possible level of ethical rigour.

### Interviews

Individuals who participated were told both the Latin and Slovene names of the plants in question and asked to provide their knowledge based solely upon that. Name was chosen for this purpose as many may have heard of the plants from family, in the media, or in books, but not had the skills to identify them by seeing a photograph or voucher specimen. For each plant, participants were asked a series of questions pertaining to general knowledge and knowledge acquisition, potential uses, as well as their natural environment and appearance.

Both types of data gathering were carried out between September 2018 and December 2019.

## Results

### Participant information

67 individuals participated in this study, with ages ranging from 18–60 with a mean age of 28 (SD = 8.45).

51 of these individuals identified their biological sex as male, while 16 identified as female. All female participants and 50 of the male participants identified as cisgender, while one of the male participants identified as non-binary.

60 of the participants defined their sexuality as heterosexual, while 3 stated they were bisexual, 2 homosexual, one pansexual, and one undefined.

63 of the 68 participants listed their nationality as Slovenian, one as French/Slovenian, and one as Serbian/Slovenian. 2 of the participants were not Slovenian (one from Russia and one from Serbia), but both live within the country. One of the participants grew up in Croatia near the border and moved to Slovenia later in life.

34 of the participants live in Ljubljana (the capital city) or the surrounding area in central Slovenia. 11 live in Gorenjska, 10 live in Primorska, 6 live in Dolenjska, one lives in Štajerska, and one lives in Prekmurje. Additionally, 2 participants are currently international students, though they are originally from Slovenia.

#### Recognition

On average, each participant believed they knew 1.6 of the 4 plants when given the Latin and Slovene names. 16 of the individuals did not recognise any of the plants by name, 17 recognised one, 15 recognised 2, 14 recognised 3, and 5 recognised all 4 plants by name.

For ease of viewing, the most frequent responses for each of the following plants have been summarised in Table 1.

**Table 1:** Comparison of most frequent responses. N = 67.

	*Atropa belladonna*	*Datura stramonium*	*Hyoscyamus niger*	*Scopolia carniolica*
Number to recognise the name	44	40	15	11
Number to have used it	3	6	1	1
Number to know people who used it	10	20	1	2
How first learned of	From family (9)	Internet (9)	University classes (5)	University classes (2)
Knowledge of plant	Toxic (12)	Toxic (5), many media cases of deaths from it (5)	Nightshade family (2), contains scopolamine (2)	Chemically similar to *A*. *belladonna* (5)
Knowledge of use	Recreational/psychoactive drug (8)	Recreational use (34)	European witchcraft (5)	European witchcraft (2)
Physical and habitat description	Blue-black-purple berries (9)	Trumpet/bell-like flowers (10)	Yellow-white/black flowers (3)	Flowers that are purple and bell-shaped/look like *A*. *belladonna* (2)
Potential misidentifications	*Solanum nigrum* (4), *Solanum dulcamara* (2)	*Brugmansia* spp. (9)		*Solanum nigrum* (1)

### *Atropa belladonna*, volčja češnja

*A*. *belladonna* was recognised by name among 44 of the 67 participants. Among these 44, 3 had used this plant before.

When asked how they had first learned about this plant, responses were varied. The most common means of hearing about this plant (accounting for 9 individuals) involved a range of responses pertaining to grandparents, parents, or older siblings warning them about the plant when they were children and telling them that it was incredibly poisonous. The next most common means, accounting for 7 of the individuals, was through a university course, either pharmacology or botany. Books accounted for 3 more while the internet was the source for 2 of the individuals. 3 had also heard about the plant through friends who had tried the plant or who knew of people who had tried it and had horrific stories about the experience. One individual knew about this plant as a result of its use in homeopathy.

In terms of what they knew about the plant, its toxicity was most commonly listed, being mentioned by 12 of the participants. 4 mentioned that it contained atropine, while an additional 2 mentioned that the fruit contained atropine. 2 stated that it had similar alkaloids to *Datura*. Only 3 individuals noted that the plant is psychoactive, while one other noted that it could cause strong hallucinations. Otherwise mentioned were that it is from the Solanaceae family, that it used to be popular in Italy, that it contains substances similar to cocaine, that the name means "beautiful woman", that it is a common plant, that it may be mistaken for blueberries, that it is an analgesic, that it can cause blindness, that it is conditionally edible, that it is a garden weed, and that it contains anticholinergic substances.

Among participants, 25 knew of uses for this plant; they listed a variety, with the most common use (listed 8 times) being as a recreational or psychoactive drug; the berries were specified for this use multiple times, though one participant also mentioned the flowers. It was then said among 6 individuals that it used to be used by witches, with one person specifying they would use the root to make ointments that they inserted into their vaginas. 5 individuals said that it used to be used cosmetically as eye drops to dilate the pupils. The next most common uses were listed 3 times, and were that the plant used to be used medicinally, or that it used to be used as a poison. Listed twice was that its berries used to be (or still are) used to strengthen alcohol, that it used to be used as a ritual sacrament in Slovenia, and that it is used in homeopathy, with one of the participants stating that it is useful for fevers. Additional items listed included that shamans used to use it to connect to wolf spirits, that it was used for treating asthma and dermatitis, that prostitutes used to use it so they would not recognise their clients, that it was an antidote to nerve gas, that it is an industrial source for the extraction of atropine, that it prevents heart ischemia, and that it is used in modern eye examinations to dilate the pupils.

Among the 67 participants, only 10 personally knew people who had made use of the plant, usually for recreational purposes, though one individual’s family uses the plant as a homeopathic medicine and one individual stated that their grandmother ate the berries when she was young as she thought they looked appealing. One individual did not know anyone personally, but had heard a story about a ritual that took place recently in Croatia where people ingested this plant and one of them fell into a fire and died.

Only 11 people claimed to know what *A*. *belladonna* looked like and/or where it grew. In terms of their comments on its appearance and habitat, participants most often commented on the berries, with 9 referring to them in hues ranging from blue to purple to black and 2 as red. 2 individuals commented that the berries are large and surrounded by leaves, 2 described the fruit as being cherry-like, and one described the fruit merely as round. 6 individuals described the plant as being a shrub, 2 that it was low-growing, one that it was a plant about 40 cm tall, one that it was a medium-sized herb, and one that it was a tree. The flowers were most often described as being white, as stated by 4 participants; listed each once were that they were trumpet-like, have flower parts of 5, have flowers like *S*. *carniolica*, and that it has red buds. The leaves were described once each as being spear-like, pointy and similar to those of *S*. *carniolica*, and being small and heart-shaped. In terms of its habitat, 3 participants noted that it grows in the forest with an additional one noting that it grows in beech forests, 2 that it grows on the edges of forests, one that it grows in the hills, and one that it grows everywhere. One noted that it grows throughout Slovenia and most of Europe, one said it grows in Europe and America, and one merely stated that it grows in Slovenia. Within Slovenia, one said it grows near the capital city and one said it grows in the Karst region.

### *Datura stramonium*, navadni kristavec

*D*. *stramonium* was recognised by name among 40 of the 67 participants. Among these 40, 6 had used this plant before, with one of them also having used the related species *D*. *innoxia*.

Among the individuals to know of this plant, the internet was the most common way of first hearing about it; this accounted for 9 individuals, with one of these specifying Erowid and one online documentaries. The next most common way of learning about this plant was through friends, with 8 of the participants first having heard of it in this manner. University courses were how 5 people found out about this plant, while books were mentioned by 4 individuals. Each stated by 3 individuals were public knowledge, knowing of people who had used it, and hearing about it in the news. Also mentioned were a trip guide, grandparents, and urban legends; additionally, one participant had learned of this plant while being told about *Brugmansia* in Peru.

In regards to what they knew of this plant, one thought that it is endemic to Slovenia, and one that it grows here both wild and as an ornamental. 3 individuals knew that it is from the family Solanaceae, 4 that it contains scopolamine, 2 that it contains alkaloids, and one that it contains anticholinergic substances. Each listed once were its use in ophthalmology, in anaesthesia, to dilate pupils, and in "mind control". 5 participants described the plant as being very poisonous while a further 2 stated that it was dangerous. One stated that only overdose on the plant is toxic, while another stated that the plant is so toxic that humans cannot safely touch it. Another participant mentioned that the plant is so dangerous that police often remove it from the cities. 5 people mentioned that it has caused some deaths over the years that have made their way into the news, with 2 of these individuals specifying cases where the victims drowned. Another participant also stated that there are many stories of people taking this plant and things going wrong. One other stated it is not a smart thing to try. Indeed, 2 participants stressed that it has severe side effects, while 3 stated that it can make you go blind; in one of these cases, it was a participant’s friend who was unable to see for nearly a week after taking the plant. It was also thought to cause permanent psychological damage by 4 participants. One individual stated it is bad for short-term memory and thus causes confusion, while another stated it is soothing to the body and slows the heart, and another that it has effects on the autonomic system. One called it a strong psychedelic and 4 others stated it was hallucinogenic, with one of them specifying the seeds as the hallucinogenic part and one stating that the hallucinations are very real and difficult to discern from reality. An additional participant stated that it can cause you to lose touch with reality. One stated that it is psychoactive, 2 that it is a deliriant, and one that it was either a deliriant or dissociative. One stated it has no spiritual use as a result of its effects, while another felt it would be easy to get the seeds on the internet. 2 individuals commented on the legality, both believing it to not be illegal in Slovenia. One noted that it has a close relative in South America that is sometimes added to ayahuasca.

37 of the participants knew of uses for this plant, with by far the most frequently listed being recreational use, stated by 34 participants. All parts of the plant were implicated in all possible manners of preparation for this use, but the most often mentioned method of doing this was by eating the seeds, as stated by 10 of these 34 participants. Among those to list the recreational usage of this plant, one said that it is used as a stimulant and one said that it is used by drug users to help with their withdrawal symptoms. The next most commonly listed uses involved medicinal applications, listed by 12 people. 3 of them stressed its use as an anaesthetic, though 2 of them noted that this is not a modern use, with one stating that pure scopolamine is now used. Listed each once were its use against coughs, nausea, bronchitis, diarrhoea, and in ophthalmology to dilate pupils. One participant also listed it as a soothing medicine while 2 noted it could be used to make a medicinal tea. One knew only that it was medicinal, while one participant specifically claimed the plant has no medicinal applications. Each listed 5 times were this plant’s use as a garden ornamental and its use in European witchcraft; among the latter, one participant elaborated that the root used to be used to make ointments that witches would apply to their vaginas with broom handles. 2 individuals stated that a tea of the plant’s seeds can induce vivid dreams, with one also stating it aids sleep. One said it is a component of "evil breath", a powder they say is used in Colombia to drug people with, while one said that when one hallucinates on this plant it will help them find other hallucinogenic plants; indeed, a further individual stressed that its traditional use was for divination. One participant said that this plant is ritually used elsewhere in the world, and another stated that it was used in the wine of ancient Greece. One person interviewed had once heard from an old woman that this plant used to be used by mothers in Slovenia to make their babies quiet, and another had heard that when planted near a vegetable garden it will keep small animals away. One knew that the plant had uses, but could not recall what they were.

Of the participants, 20 knew someone who had used this plant before. The stories invariably involved terrible trips with long-lasting effects. One person even said they had a friend who continued having hallucinations for 2 weeks after using the plant. One person had a friend who had sold the seeds before. Also mentioned were knowing someone who drowned after taking it and knowing someone who took it and then woke up naked in a ditch in the neighbouring country of Hungary. 2 people mentioned that their friends had permanent damage done by this plant, one stating that it triggered psychosis and one that their friend who took it has never been quite right since.

31 of the participants claimed to have some knowledge of *D*. *stramonium*’s appearance or growing habits. The flowers were the most frequently commented on trait, with it being stated 10 times that they have trumpet or bell-like flowers, 9 times that the flowers are white, 2 times that the flowers are yellow, and once each that the flowers are blue-red or that they may be in a range of colours including blue, white, and purple. 4 individuals noted that it produces a spikey fruit, one that it grows its seeds in pods, and one that it has spikes somewhere on the plant. The leaves were only described twice, once each as being tomato-potato-like and as being toothed. 2 individuals said it was a shrub, with one emphasising that it is fairly tall and the other that it is dark green. One individual noted it is about one metre tall, while another stated that it grows and blooms in summer. In terms of where the plant is from, 2 participants listed South America, one listed central Asia, one listed Latin America and the Middle East, one listed Africa, and one listed the Mediterranean coast. One individual stated that it grows in Croatia, but not in Slovenia. For those who said that it grows in Slovenia, 2 noted that it is a weed that grows in fields and near roads, one said that it usually grows in gardens, one said that it usually grows in the forest, and one that it grows in front of the police station in Ljubljana. While one individual stated that it tends to grow around old houses, another stated that it likes to grow in the disturbed earth around new buildings. One individual stated that it grows in the limestone Karst region of the country.

### *Hyoscyamus niger*, črni zobnik

*H*. *niger* was recognised by name among 15 of the 67 participants. Among these 15, one had used this plant before.

For participants who knew of this plant, university classes were the most frequent way of first being introduced to it, being listed by 5 individuals, one specifying botany classes and one specifying pharmacognosy classes. The internet was listed by 4 individuals while books were listed by 3.

In terms of what they knew about this plant, 2 individuals stated that it is in the nightshade family. One said that hyoscyamine is named after this plant, while 2 mentioned that it contains scopolamine. Each mentioned once were that it causes terrible trips, that it is poisonous and hallucinogenic, that it is psychoactive, that it is a deliriant, and that it contains alkaloids. One individual also described the plant as having a gentle yet masculine spirit.

When listing the ways in which this plant is used, the most commonly stated was that it used to be used in European witchcraft; 5 individuals stated this, with one specifying that witches would make an ointment of the root that they would apply to their vaginas with a broom handle. One said that the plant used to be used in ointments in Slovenia. 3 individuals stated it to be medicinal, with one specifying that it works as an anaesthetic. 3 participants said it can be used as a recreational substance, with one specifying it works as a hallucinogen. One of these participants said the leaves of the plant can be made into an ointment with *A*. *belladonna* and applied to the skin to achieve hallucinogenic effects.

Among the participants to know of this plant, only one knew someone who has actually used it. This participant is also the one who had previously used it themself. One participant noted that though they do not personally know anyone who took this plant, they did watch a documentary in which 2 individuals take it.

8 participants claimed to know about the appearance or habitat of *H*. *niger*. Most often commented on were the flowers, which were variously said to be small, yellow-white, blackish, and yellow. The leaves were the next most commented on, being variously stated to be dark brown, yellow, to have serrated edges, and to be hairy. Indeed, one individual stated that the whole plant is hairy; another stated it is a small bush. One individual said they could recognise it in photos but did not know it well enough to describe it. 2 individuals said that it grows in Slovenia, one stating that it grows in the forest and one that it is rare here.

### *Scopolia carniolica*, kranjska bunika

*S*. *carniolica* was recognised by name among 11 of the 67 participants. Among these 11, 2 had used this plant before.

For how they had first heard of this plant, 2 participants stated they heard about it through university classes, with one specifying that it was due to botany classes. One first heard about it through books, and one first heard about it from an acquaintance.

2 participants commented on this plant’s family affiliation, one noting it falls in the nightshade family and one noting it is a member of the Solanaceae. 5 individuals stated that this plant is chemically similar to *A*. *belladonna* while 2 said it is chemically similar to *Datura*. 2 individuals said it contained scopolamine, one that it contained atropine, and one that it contained both atropine and scopolamine. One noted that it has a high alkaloid content. One participant stated that this plant was first described by the botanist Scopoli, while another noted that it might be hallucinogenic. One participant said that the plant is said to keep evil spirits away.

In terms of uses for this plant, 2 individuals noted that the rhizome used to be used by witches to make an ointment that they applied to their vaginas to help them fly. One stated that it is used in modern pharmaceuticals, and one that it used to be used as a medicine. One said that the leaves are tied around the waist of people in order to protect them from evil spirits.

Only one individual knew of others who had taken this plant, and they stated they know of a couple of cases of people taking it and then ending up in psychiatric hospitals for a while.

7 participants claimed to know what this plant looks like or how it grows. 2 commented on the flowers, one saying they are like those of *A*. *belladonna* and one that they are bell-shaped and of a purple colour. 2 commented on the leaves, one saying they are pointy and the other stating they are a noticeable green. One individual commented on its underground rhizome, while one noted that it is a low-growing plant. One said it is only found in the area around Tolmin and Idrija, while another said that it grows in forests in dolomitic areas.

## Discussion

### Knowledge acquisition

In terms of the number of plants participants were able to identify by name, there was a fair degree of similarity between knowing 1, 2, or 3 plants, but with a large drop in numbers for those claiming to know all 4 of the plants, with less than 8% of the study group stating they recognised all of the plants under investigation (meanwhile, about 3 times as many people were unable to identify any of the plants). Those who tended to have the most detailed knowledge about these plants and to recognise the largest number by name were those who had undertaken university level studies and had courses in botany or pharmacognosy where they had learned of these plants, suggesting that "traditional" knowledge has been greatly eroded and largely replaced with academic learning when it comes to these plants. Indeed, a small number of people were even able to name the family in which these plants are classified and a fair few were able to comment to varying degrees of specificity on the alkaloids that they are best known for. This not only displays a shift from "traditional" to "modern" knowledge acquisition, but also highlights the shifting nature of the knowledge itself from applied to academic; where once use knowledge and skills would have prevailed, factual knowledge divorced from practicality has largely come to take its place. One of the few facts to be passed down "traditionally" through generations about these plants were their toxicity rather than their wealth of uses. Indeed, the spread of Western medicine and the globalised movement of recreational drugs has made many of the uses of these plants essentially obsolete, especially given their potentially dangerous side effects.

### Differences between the investigated plants

**Fig 1:**
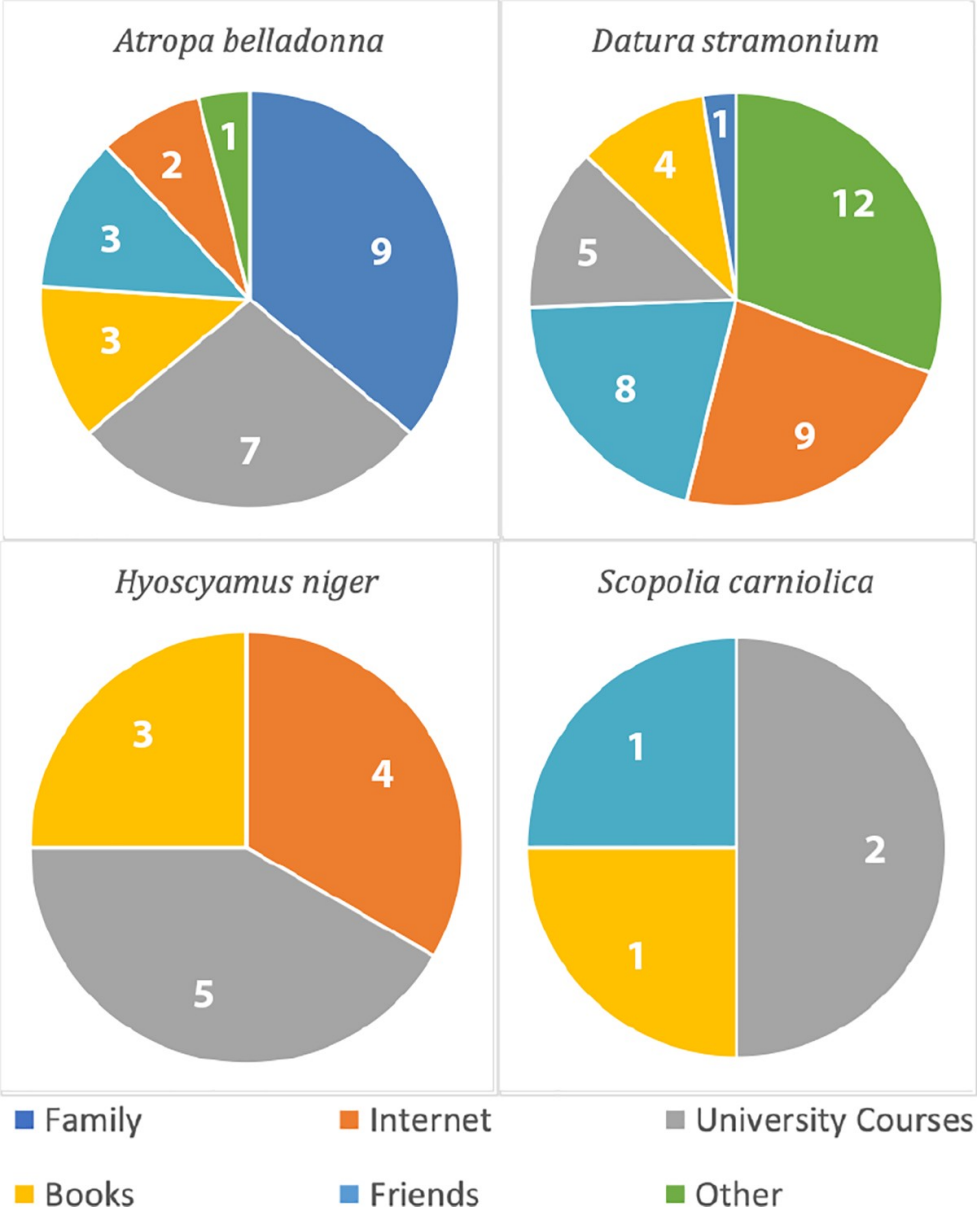
Methods of learning by plant.

Even between the plants there were clear differences in how individuals had learned about them. *A*. *belladonna* was the only plant to have a large number of individuals state that they learned about it from family members, perhaps suggesting that this is the only of these plants that is being passed down along "traditional" channels in any significant way. University courses were not far behind as a source of learning about this plant, displaying another important source of information. For *D*. *stramonium*, the internet was the main source of knowledge, unsurprising as this plant is used globally as a recreational drug among youth. *H*. *niger* and *S*. *carniolica* were both mainly learned of through university courses, suggesting that knowledge of these plants has been largely lost in intergenerational transmission and that they have not obtained the same level of infamy in modern times as their two previous relatives. A visualisation may be seen in Fig 1.

The most recognised as a recreational substance was *Datura stramonium*, being mentioned by 34 participants. *Atropa belladonna* was mentioned in this context only 8 times, *Hyoscyamus niger* 5 times, and *Scopolia carniolica* was not mentioned as a recreational substance at all, despite containing the same alkaloids. This also brings about the question as to the difference between recreational and other uses, as two people had made use of *S*. *carniolica*, though clearly neither felt they had done so in a recreational manner. As previously mentioned, knowledge of *D*. *stramonium* as a recreational substance is a much more global trend, and thus the spread of knowledge of this plant likely is the result of media rather than an intergenerational sharing of knowledge. One of the few obvious examples of "traditional" knowledge in this study, however, is related to *D*. *stramonium*, where the participant explicitly stated that they had been informed by an old woman that people in Slovenia used to use *D*. *stramonium* to make their babies quiet. Even after extensive literature reviews on the uses of these plants in Europe, this was the first mention I have found to such a practice. This could suggest a previously undocumented use, or point to an inherent danger in the "traditional" passing down of knowledge: all those involved must be speaking about the same thing, have obtained the knowledge from a credible source, and have correctly remembered and recalled it. This plant was also said to be used around gardens to keep pests away, similar to its known uses to keep pests away from hen houses in Italy [32].

### Errors in identification

Knowledge was not infallible in this study; many individuals described plants that were quite obviously not those being researched. 6 people described *A*. *belladonna* in such a way as to be obvious that they were referring to a different plant or that they at least were mistaken about some of its features. 4 individuals mentioned white flowers, possibly referring to *Solanum nigrum*; indeed, this was often combined with stating it has black berries, another trait of the species. Despite their physical differences, these two plants have previously been reported as being frequently mistaken for each other [33]. 2 mentioned red berries, perhaps thinking of *Solanum dulcamara*. As both plants are common weeds, it is unsurprising that they would come to mind for some. *Solanum nigrum* likewise posed a problem for one of the individuals who thought they knew *S*. *carniolica*; their description seemed to be of *Solanum nigrum*, and upon seeing a picture of this plant they confirmed that they believed it to be *S*. *carniolica*.

During interviews, it became apparent that at least 9 individuals to recognise *Datura stramonium* by name were actually speaking about *Brugmansia spp*. Though this genus is closely aligned and was once also classified in *Datura*, it is now considered distinct. Besides being visually different, it also has a different Slovene name (angelska trobenta). For those who did not seem to certainly be speaking about *D*. *stramonium*, they were asked if the flowers on the plant point upwards or downwards. Though *D*. *stramonium* flowers point upwards, *Brugmansia spp*. flowers hang like pendants from the plant. This confusion could be a result of a general lack of botanical knowledge, or of the newer system not having been as widely accepted in Slovenia with the common names then coming to be confused. The frequent terminological mixing between words such as herb, shrub, and tree, for example, also serves to display the potential ambiguity of folk classifications, as does the description of *A*. *belladonna* here given where it was said to have "leaves" surrounding the berry when in fact the participant was likely referring to the prominent sepals.

### Erroneous information

In addition to obvious issues of identification, a great deal of non- or pseudo-factual information was stated as fact. For example, many participants mentioned that these plants were once used as ointments applied to the vaginas of European witches with brooms. Though this is indeed a widely held belief, it is not actually proven that such ointments were used, and even less proven that they were used by being introduced into the vagina [34]. This, however, does not show that "traditional" knowledge was more accurate than its "modern" incarnation; for centuries people believed that these plants were used along with the fat of dead babies and other putrid ingredients to allow witches to literally fly through the air and travel to their sabbath meetings where they would fornicate with the devil, a "traditional" notion that most of us today would deem absurd. The repetition of "fact" leads to it being incorporated into traditional knowledge; one participant noted that *D*. *stramonium* was used to strengthen wine in ancient Greece. Indeed, many books and even academic papers today make claims about the use of *D*. *stramonium* in Classical and Medieval Europe, despite the fact that botanists are largely in agreement that this plant arose in the Americas and would not have made its way to Europe until the 1500s [11, 35]. Such incorporation of incorrect information could be happening throughout "traditional" knowledge systems at each step of their transmission, once more showing that simply being "traditional" does not make knowledge accurate.

One participant mentioned that *A*. *belladonna* had been ritually used by shamans to contact wolf spirits, though this is also not proven; this belief was likely stated as the Slovene name (volčja češnja) literally translates to "wolf cherry". Many urban legends were also mentioned, in which individuals did not know people who had used these plants, but "knew of" people having used these plants, often dying or having serious negative consequences. Incorrect terms such as "psychedelic" were also used to described these plants, perhaps displaying that this term and the term "hallucinogenic" are equal in the minds of many. Additionally, though these plants are undeniably dangerous and toxic, the degree of their toxicity was often exaggerated, for example where one individual said *D*. *stramonium* is removed by police in the capital city due to its toxicity; as many plants that are far more poisonous grow all around the city, it seems more likely that this practice (if real) is carried out due to the high rate of abuse of this plant among young people. As knowledge of toxicity was the main element "traditionally" passed down through family lines, this may suggest that knowledge obtained from close personal contacts is given a prominent place in our minds, or may simply point to the role of the repetition of information during the formative years of our youth.

### Assumed clarity

It is also worth noting that in many cases participants may have failed to provide information, thinking that their other responses implied it. For example, more individuals stated that these plants can be used as recreational substances than that they are psychoactive. Whether this reflects an assumption that stating a plant can be used recreationally means it is psychoactive or that the individuals were unfamiliar with the concept of psychoactivity is unclear.

### The role of the internet

Though ethnobotanical fieldwork has historically been based upon field interviews, participants in the present research were given the option to be interviewed in person or to complete an online questionnaire. Just as "traditional" knowledge evolves, so too do societal norms, and research methods must also grow to keep pace. As in-person interviews and field work were not necessary for this type of research, it allowed a greater number of individuals to participate, many of whom would not have elected to do so in a face-to-face interview for reasons of privacy or convenience. This impersonal form of data gathering may also have served to lessen social desirability bias in responses, though also limited the interviews to a very structured format. Overall, approximately 60% of participants elected to fill out a questionnaire rather than participating in a live interview.

The mentioning of Erowid (a harm-reduction web resource that also offers an index of pertinent web forums) also brings up the question of how knowledge is transmitted through the internet. With web forums such as Reddit being very popular, it further raises the issue of when knowledge is "traditional" or not. In using web forums, individuals are not merely reading about information written by a source, but actively interacting with another individual. This essentially mirrors the intergenerational passage of knowledge within families. Yet most would not consider knowledge gained through a web forum to be "traditional". If a person’s grandmother were to teach them about traditional uses of plants through such a medium, would it be more or less traditional than getting the information from another person? We all have our own traditions, and the notion of "traditional" knowledge needs further investigation and consideration within the field of ethnobotany, especially as it applies to the modern spread of knowledge through resources that were unavailable (and, indeed, unimaginable) to previous generations.

## Conclusion

Overall, knowledge of these plants was very lacking among users of hallucinogenic plants and mushrooms in Slovenia, showing that the "traditional" use of these plants as recreational substances has declined. Each plant was unique: *Atropa belladonna* was the most recognised by name, but *Datura stramonium* was the plant that was most used; *Scopolia carniolica* was the least recognised plant, while *Hyoscyamus niger* was the least used. Though many offered reliable descriptions of the plants, a fair number gave clearly erroneous information, often likely referring to other Solanaceae plants. Though this may display a lack of specific botanical knowledge, it may be indicative of a higher level of general botanical knowledge; the plants that were confused with those studied here have very typical Solanaceae flowers, and it is possible that the individuals to make these mistakes were making the correct family association as a result of this but failing to connect to the correct species. This may be due to the greater similarity of the flowers of these common weeds to those of common Solanaceae garden plants, which many more people are likely to recognise. The flowers on the species here studied, however, are less immediately recognisable as being from the Solanaceae to the untrained eye.

Though the knowledge of the uses of these anticholinergic Solanaceae plants is clearly not very detailed among hallucinogenic plant and mushroom users in Slovenia, some does exist, largely arising from education and use of the internet rather than oral legacies passed through families. Though largely dangerous when used as recreational substances, some tradition and knowledge surrounding these plants has been passed down. Indeed, increased education about these plants and their traditional uses may be valuable, if for no other reason than to ensure youths are aware of the dangers these plants pose.

## References

[1] Carruthers DMJ. Lines of Flight of the Deadly Nightshade: An Enquiry into the Properties of the Magical Plant, its Literature and History. Mosaic a J Interdiscip study Lit. 2015;48(2):119–32.

[2] Porta G. Natural Magick. London: Thomas Young & Samuel Speed; 1658.

[3] Ruck CAP. Entheogens in Ancient Times In: Wexler P, editor. History of Toxicology and Environmental Health: Toxicology in Antiquity, Volume II London: Elsevier; 2015 p. 116–25.

[4] Stika HP. Traces of a possible celtic brewery in eberdingen-hochdorf, kreis ludwigsburg, southwest Germany. Veg Hist Archaeobot. 1996;5(1–2):81–8.

[5] Stevanović ZD, Petrović M, Aćić S. Ethnobotanical Knowledge and Traditional Use of Plants in Serbia in Relation to Sustainable Rural Development In: Piernoi A, Quave CL, editors. Ethnobotany and Biocultural Diversities in the Balkans. New York: Springer; 2014 p. 229–52.

[6] Dioscorides. De Materia Medica. Johannesburg: Ibdis Press; 2000.

[7] Hocking GM. Henbane-Healing Herb of Hercules and of Apollo. Econ Bot. 1947;1(3):306–16.

[8] Pliny the Elder. The Natural History. London: Taylor and Francis; 1855.

[9] Van Bingen H. Physica: Of Various Natural Creatures, The First Book Concerning Plants. Boston: Beacon Press; 2001.

[10] Gunda B. Fish Poisoning in the Carpathian Area and in the Balkan Peninsula. Kroeber Anthropol Soc Spec Publ. 1967;1:1–33.

[11] Daunay M, Laterrot H, Janick J. Iconography of the solanaceae from antiquity to the XVIIth century: A rich source of information on genetic diversity and uses. Acta Hortic. 2007;745(May):59–88.

[12] Penicka S. Caveat Anoynter! A Study of Flying Ointments and Their Plants. Dark Side Proc SeventhAustralian Int Relig Lit Arts Conf [Internet]. 2008;181–95. Available from: http://openjournals.library.usyd.edu.au/index.php/SSR/article/viewFile/210/189

[13] Aikman J. The History of Scotland, Translated from the Latin of George Buchanan; with Notes and a Continuation to the Union in the Reign of Queen Anne. Vol. I Glasgow: Blackie, Fullarton, &. Co.; 1827.

[14] Fatur K. Sagas of the Solanaceae: Speculative ethnobotanical perspectives on the Norse berserkers. J Ethnopharmacol [Internet]. 2019;244(June):112151 Available from: 10.1016/j.jep.2019.112151 31404578

[15] Fatur K. “Hexing Herbs” in Ethnobotanical Perspective: A Historical Review of the Uses of Anticholinergic Solanaceae Plants in Europe. Econ Bot. 2020;74(2):140–58.

[16] Arroo RRJ, Woolley JG, Oksman-Caldentey KM. Tropane alkaloid containing plants—Henbane, Belladonna, Datura, and Duboisia In: Nagata T, Lörz H, Widholm JM, editors. Transgenic Crops VI, Section II: Medicinal Crops, Biotechnology in Agriculture and Forestry Vol 61 Berlin: Springer-Verlag; 2007 p. 2–20.

[17] Maheshwari NO. Rediscovering the medicinal properties of Datura sp.: A review. J Med Plants Res [Internet]. 2013;7(39):2885–97. Available from: http://www.academicjournals.org/journal/JMPR/article-full-text-pdf/75DA4CE41227

[18] Merlin MJ. Archaeological Evidents for the tradition of psychoactive plant use in the old world. NY Bot Gard. 2003;57(3):295–323.

[19] Piccillo GA, Miele L, Mondati E, Moro PA, Musco A, Forgione A, et al Anticholinergic syndrome due to “Devil’s herb”: When risks come from the ancient time. Int J Clin Pract. 2006;60(4):492–4. 10.1111/j.1368-5031.2006.00864.x 16620365

[20] Waniakowa J. Mandragora and Belladonna—the Names of Two Magic Plants. Stud Linguist Univ Iagellonicae Cracoviensis. 2007;124:161–73.

[21] Cilenšek M. Naše škodljive rastline v podobi in besedi [Internet]. Klagenfurt: Družba sv. Mohorja v Celovcu; 1892 [cited 2019 Mar 28]. Available from: https://sl.wikisource.org/wiki/Naše_škodljive_rastline/Škodljive_človeku_in_živini

[22] Díaz JL. Sacred plants and visionary consciousness. Phenomenol Cogn Sci. 2010;9(2):159–70.

[23] Sanz C, Tagliazucchi E. The experience elicited by hallucinogens presents the highest similarity to dreaming within a large database of psychoactive substance reports. Front Neurosci. 2018;12(JAN):1–19. 29403350 10.3389/fnins.2018.00007 PMC5786560

[24] Tchórz M, Dobryniewska W, Fałkowska U, Radzka A, Krawiec K. Evaluation of knowledge of Polish medical students regarding toxic plants. Polish J Public Heal. 2018;128(1):19–25.

[25] Leonti M. The future is written: Impact of scripts on the cognition, selection, knowledge and transmission of medicinal plant use and its implications for ethnobotany and ethnopharmacology. J Ethnopharmacol [Internet]. 2011;134:542–55. Available from: 10.1016/j.jep.2011.01.017 21255636

[26] Lenclud G. La tradition n ‘est plus ce qu’elle était… Sur les notions de tradition et de société traditionnelle en ethnologie. Terrain. 1987;9:1–14.

[27] Tareau MA, Palisse M, Odonne G. As vivid as a weed… Medicinal and cosmetic plant uses amongst the urban youth in French Guiana. J Ethnopharmacol [Internet]. 2017;203:200–13. Available from: 10.1016/j.jep.2017.03.031 28347829

[28] Baraniecka-Olszewska K. Re-enacting Historical Slavic Rites in Contemporary Poland: The Rekawka Fair in Cracow. Anthropol J Eur Cult. 2016;(27):118–35.

[29] MIVILUDES. Rapport au Premier ministre [Internet]. 2007 [cited 2019 Mar 29]. Available from: https://www.derives-sectes.gouv.fr/sites/default/files/publications/francais/Rapport_Miviludes_2007.pdf

[30] Fatur K. Duhovi Rastlin, Duša Stare Vere: The Use of Plants in Sacred Rituals Among Nature Worshippers in Slovenia. The Pomegranate. 2018;(Awaiting Publication).

[31] Fatur K. Peculiar plants and fantastic fungi: An ethnobotanical study of the use of hallucinogenic plants and mushrooms in Slovenia. PLoS One [Internet]. 2021;16(1):e0245022 Available from: 10.1371/journal.pone.0245022 33412556 PMC7790546

[32] Leporatti ML, Guarrera PM. Ethnobotanical Remarks in Capitanata and Salento Areas, Southern Italy. Ethnobiologica. 2007;64(2005):51–64.

[33] Slaughter RJ, Beasley MG, Lambie BS, Wilkins GT, Schep LJ. Poisonous plants in New Zealand: a review of those that are most commonly enquired about to the National Poisons Centre. J New Zeal Med Assoc NZMJ [Internet]. 2012 [cited 2019 Mar 27];125(1367):87–118. Available from: https://www.nzma.org.nz/__data/assets/pdf_file/0007/36493/slaughter.pdf 23321887

[34] Ostling M. Babyfat and Belladonna: Witches’ Ointment and the Contestation of Reality. Magic, Ritual Witch. 2016;11(1):30–72.

[35] Geeta R, Gharaibeh W. Historical evidence for a pre-Columbian presence of Datura in the Old World and implications for a first millennium transfer from the New World. J Biosci. 2008;32(S3):1227–44. 10.1007/s12038-007-0132-y 18202447

